# Retinitis linked to human herpesvirus type 6: a case study in a splenectomised patient

**DOI:** 10.1186/s12348-025-00460-2

**Published:** 2025-02-10

**Authors:** Joana Santos-Oliveira, Ana Maria Cunha, Ana Filipa Moleiro, Margarida Ribeiro, Sónia Torres-Costa, Cláudia Oliveira-Ferreira, Ana Catarina Pedrosa, Joana Araújo, Luís Figueira, Marta Silva

**Affiliations:** 1 Department of Ophthalmology, Centro Hospitalar Universitário de São João, Porto, 4200–319 Portugal; 2 Department of Surgery and Physiology, Faculty of Medicine of University of Porto, Porto, Portugal; 3 Pharmacology and Therapeutics Unit, Department of Biomedicine, Faculty of Medicine of University of Porto, Porto, Portugal

## Abstract

Human herpesvirus-6 (HHV-6) is a member of the HHV family and is a rare cause of infectious uveitis. We report a case of a splenectomised patient, hospitalised due to invasive pneumococcal disease, who was diagnosed with retinitis in the right eye, with good visual acuity (0.1 LogMAR). Given the presence of HHV-6 Polymerase chain reaction (PCR) in the cerebrospinal fluid and the serum and the coexistence of a severe central nervous system infection, the ophthalmological features were attributed to the HHV-6 infection. He was treated with topical corticosteroid eyedrops, cyclopentolate, and prednisolone acetate ointment and systemically with intravenous ganciclovir 5 mg/kg bid for 14 days and then oral valganciclovir 900 mg bid for 4 weeks. The diagnosis was promptly presumed, enabling the early initiation of appropriate treatment and contributing to the preservation of the good visual acuity initially observed.

## Introduction

Human herpesvirus-6 (HHV-6), originally designated human B-lymphotropic virus, is a member of the HHV family and was isolated from cultured lymphocytes from individuals with lymphoproliferative disorders in North America, in 1986 [1].

HHV-6 has two variants (6A and 6B), which can be distinguished by serological characteristics, genetic features, and host cell range. The B variant is usually responsible for the primary infection that generally occurs by the age of 2 years and is the main cause of *roseola infantum*, also referred to as *exanthema subitum*. Primary infection in healthy adults is rare [2–4].

Like all herpesviruses, HHV-6 establishes life-long latency in human hosts. Reactivation of a latent infection can cause serious complications, especially in immunocompromised patients, such as bone marrow or solid organ transplant recipients [4, 5].

Intraocular inflammation associated with HHV-6 infection is rare, likely underdiagnosed, and more commonly seen in immunocompromised patients. It can involve neuro-ophthalmological disorders, such as optic disc neuropathy, corneal endotheliitis, and uveitis, with the most common form being posterior uveitis resembling acute retinal necrosis. Both variants 6A and 6B have been associated with cases of intraocular inflammation [6–15].

Splenectomy per se increases the risk of infection to encapsulated bacteria, Gram-negative pathogens, and intra-erythrocyte parasites [16].

In the present study, we report a case of a splenectomised patient, hospitalised due to invasive pneumococcal disease, who was diagnosed with retinitis in the right eye (OD). HHV-6 DNA was detected both in serum and in cerebrospinal fluid (CSF).

## Case report

A 41-year-old Caucasian Portuguese man, who had undergone splenectomy following a car accident, was hospitalised at our center with invasive pneumococcal disease presenting as meningitis/ventriculitis and bacteraemia caused by *Streptococcus pneumonia*. Apart from splenectomy, the patient was healthy and did not take any usual medication. He had no other risk factors for infectious disease: he lived in an apartment, had no contact with animals, did not consume unpasteurized dairy products nor untreated water, did not carry out significant activities in rural areas, had not travelled abroad recently, nor had any recent contact with diseased people. However, he had not received immunization against encapsulated bacterial pathogens.

His lumbar puncture analysis by polymerase chain reaction (PCR) was positive for *Streptococcus pneumonia* and for HHV-6, having been identified 370.000 copies/mL, and was negative for herpes simplex virus type 1 and 2 (VSH1 and VSH2), varicella-zoster virus, toxoplasma gondii, and cytomegalovirus (CMV). These microorganisms are part of the standard panel tested for meningitis and encephalitis cases at our hospital.

He was treated with intravenous ceftriaxone (for 8 days) and dexamethasone (for 4 days), with good clinical and analytical evolution.

On the 11th day of hospitalization, the patient developed redness in the OD and reported blurred vision, prompting a consultation with the ophthalmology department. He had no previous history of eye disease.

In the OD, the best-corrected visual acuity (BCVA) was 0.1 LogMAR and in biomicroscopy he presented ciliary hyperemia, fine keratic precipitates and 2 + cells in the anterior chamber (The Standardization of Uveitis Nomenclature - SUN criteria) [17]. In the left eye (OS), the BCVA was 0.0 LogMAR and the slit-lamp examination revealed no abnormalities. Intraocular pressure was 5 mmHg in OD and 9 mmHg in OS (Goldman applanation tonometry). OD dilated fundus examination exhibited mild vitreous haziness, and a well-perfused and defined optic disc with a normal cup-to-disc ratio. The macula presented hemorrhages near the superior temporal arcade and in the peripheral nasal retina there was a creamy lesion with about two optic discs in diameter, with ill-defined edges (See pictures 1 A and 1B). Several hemorrhages and exudates were present around this retinal lesion. No pigmentary cicatricial lesion was found suggestive of a past toxoplasmosis infection, nor signs of vasculitis. In OS dilated fundus examination was normal. Macular OCT examination did not present any significant change, including signs of edema (See pictures 2A and 2B).

**Picture 1:**
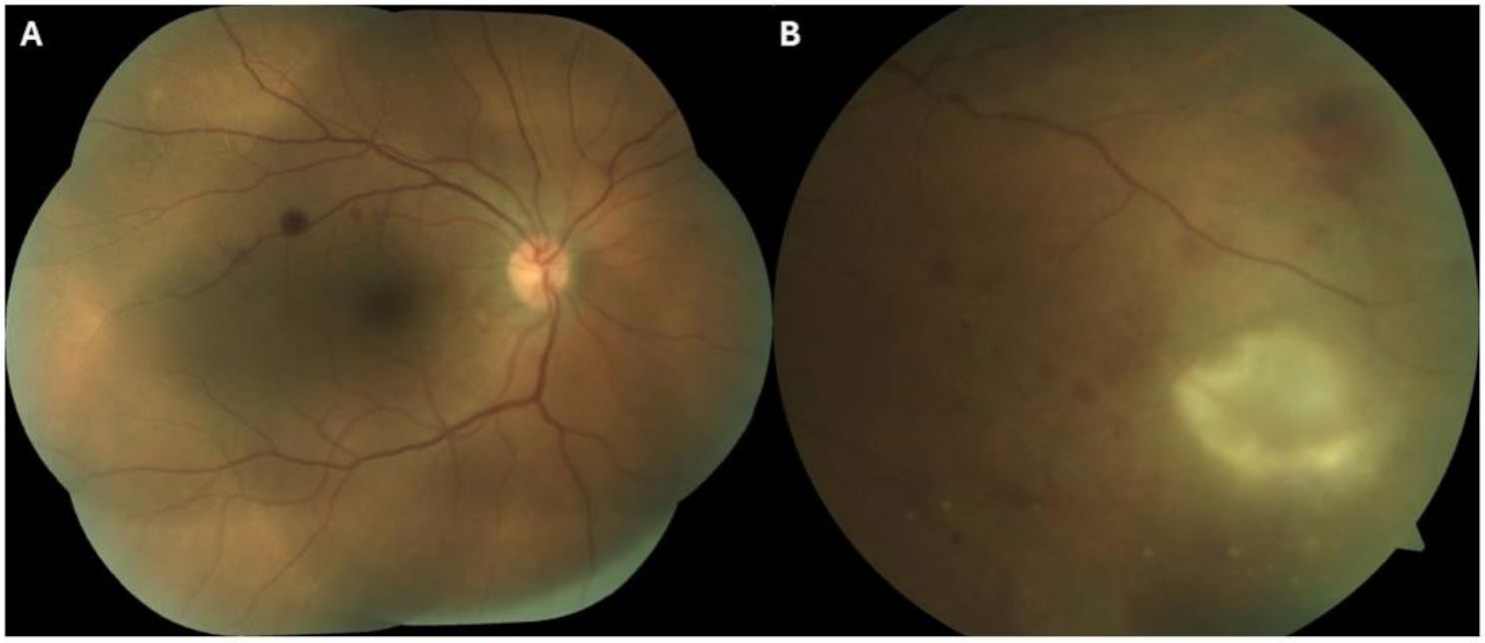
(**A**) OD - The macula presented hemorrhages near the the superior temporal arcade; (**B**) In the nasal periphery, a creamy lesion was identified with about two optic discs in diameter, with ill-defined edges, and several hemorrhages around it

**Picture 2:**
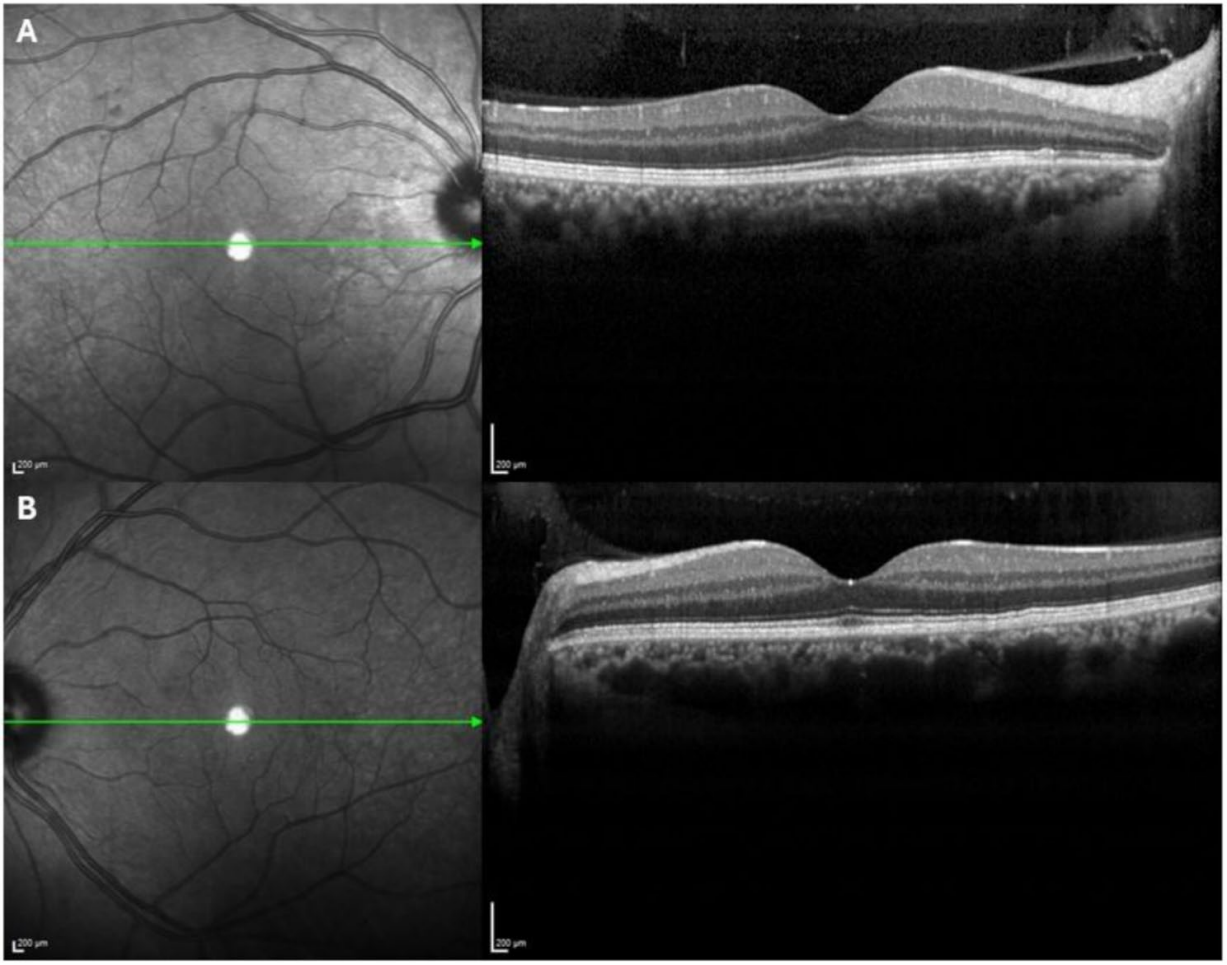
OCT macular exam OD (**A**) and OS (**B**)

Given the presence of HHV-6 PCR in the CSF and the coexistence of a severe central nervous system (CNS) infection, the ophthalmological features were attributed to the HHV-6 infection.

He started treatment with topical corticosteroid (dexamethasone phosphate 1 mg/mL tid), cyclopentolate 10 mg/mL bid, and prednisolone acetate ointment 5 mg/g at night. Systemically, he started intravenous ganciclovir 5 mg/kg bid.

Serological PCR testing was positive for HHV-6 (24.000 copies/ml). Syphilis and human immunodeficiency virus (HIV) tests were negative, and serologic antibody test results of CMV, VHS1, VHS2, toxoplasma gondii, and Epstein-Barr virus (EBV) can be seen in Table 1.

**Table 1:** Serologic antibodies test results

	IgM	IgG
CMV	Negative	**Positive**
VHS-1	Negative	**Positive**
VHS-2	Negative	Negative
Toxoplasma gondii	Negative	Negative
EBV	Negative	**Positive**

The patient evolved favorably with the recommended treatment, so no further invasive exam was proposed (e.g. diagnostic vitrectomy or anterior chamber tap).

Intravenous ganciclovir 5 mg/kg bid was continued for 14 days and then substituted with oral valganciclovir 900 mg bid for 4 weeks.

He was discharged on the 4th day of valganciclovir treatment, with the indication to continue oral therapy at home. Vaccination against capsuled bacteria was scheduled. In a follow-up consultation, one month after being discharged, serological PCR testing for HHV-6 was still positive, but with a reduced number of copies/mL (7600 copies/ml).

In the first ophthalmology follow-up consultation, 2 months after being discharged, he presented asymptomatic, having completed the treatment posology. BCVA was 0.1 LogMAR in OD and 0.0 LogMAR in OS. Biomicroscopy in both eyes did not present any signs of inflammation. Dilated fundus examination in OD revealed no vitritis, the optic disc was well-perfused and defined, the macula was unremarkable, and the retinal lesion presented signs of cicatrization, with only one visible haemorrhage in the area (See Picture 3 A and 3B).

**Picture 3 OD -:**
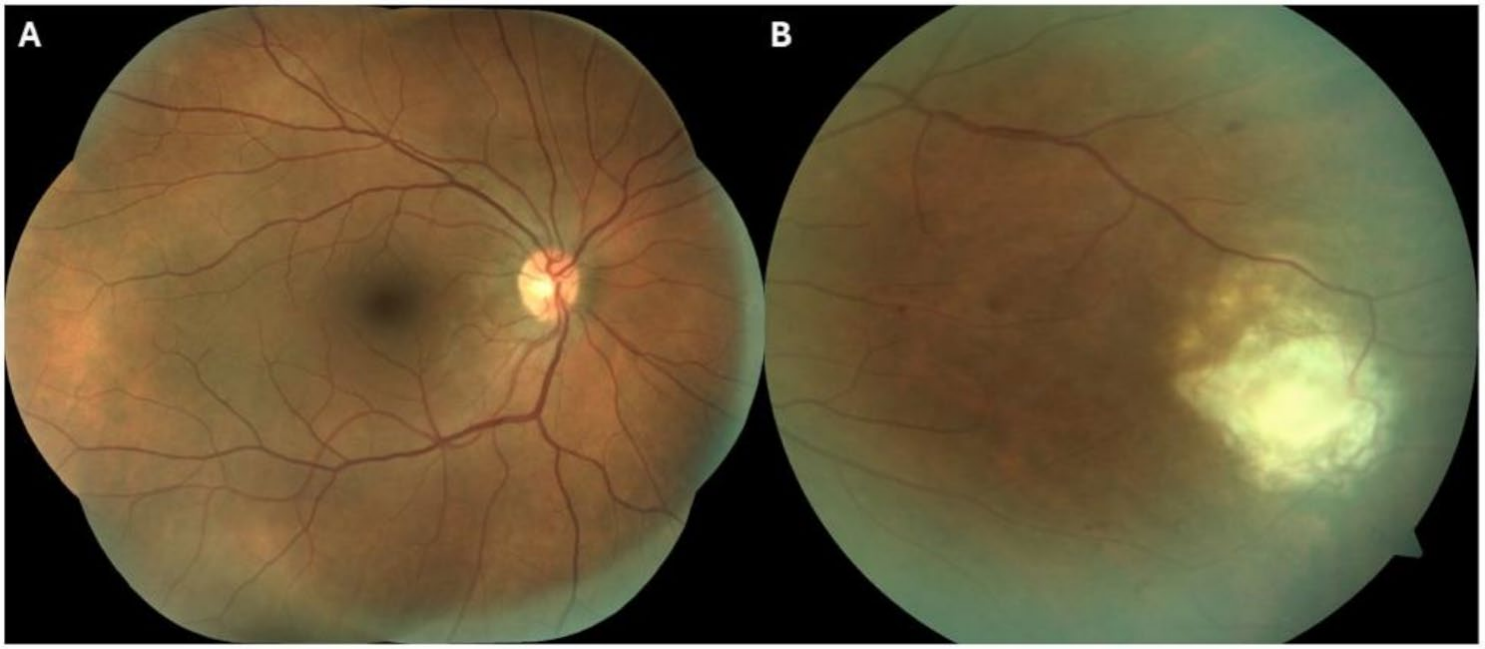
(**A**) Posterior pole without hemorrhages. (**B**) In the nasal periphery, the retinal lesion appeared to be less active, with fewer hemorrhages in the area

One year and a half after the diagnosis, the patient was asymptomatic, without any treatment and presented BCVA of 0.1 LogMAR OD and of 0.0 LogMAR OS. Regarding the OD, there were no signs of ocular inflammation, the disc and macula appeared normal, and a cicatricial scar was observed in the nasal retina (See Picture 4 A and 4B). OS remained completely normal. The patient was discharged from our department, with an indication for regular ophthalmology follow-up.

**Picture 4 OD -:**
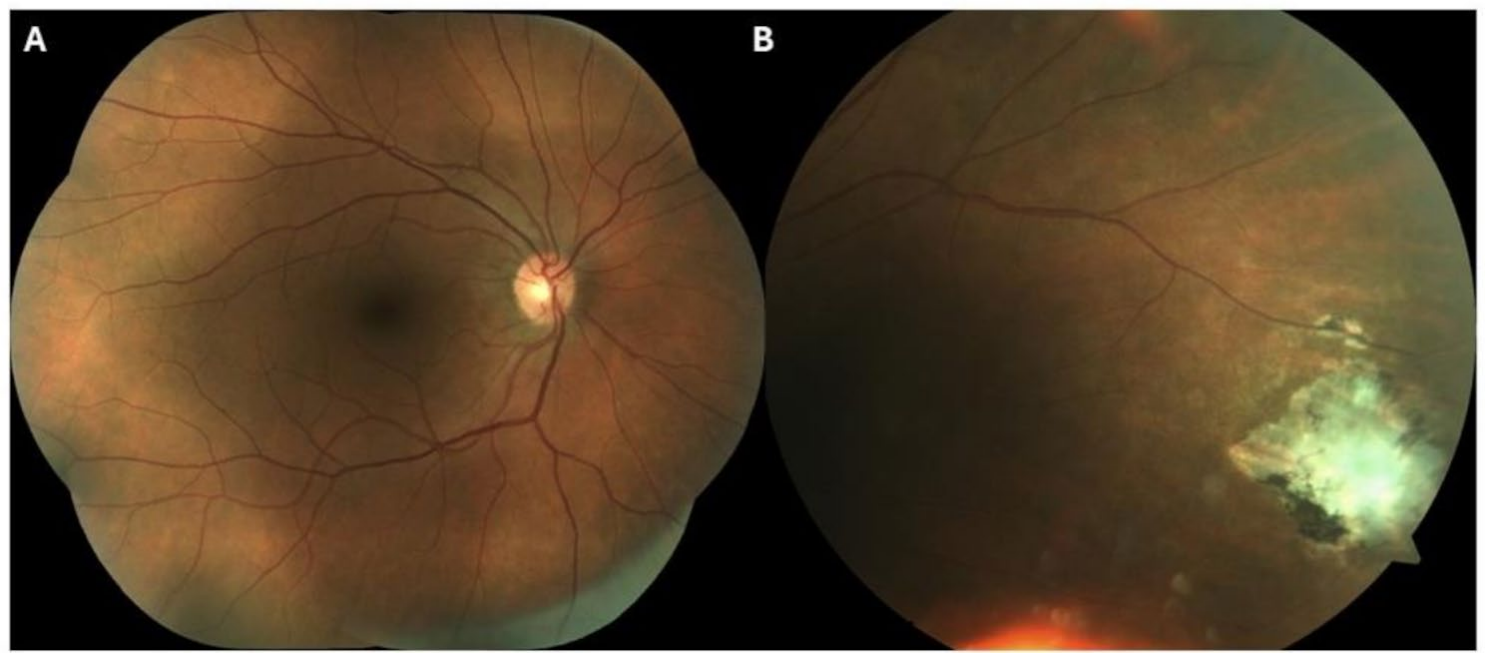
(**A**) Optic disc and macula unremarkable. (**B**) In the nasal periphery, the retinal lesion presented signs of cicatrization

## Discussion

HHV-6 is a rare cause of infectious uveitis, which can present in various forms, including corneal endotheliitis, posterior uveitis (more frequently as acute retinal necrosis), panuveitis, or optic neuritis [6–15].

In our report, we describe a case of unilateral retinitis in a splenectomised patient, hospitalized due to invasive pneumococcal disease.

Before this episode, our patient did not present any risk factors for infection other than being splenectomised. Following splenectomy, individuals have an elevated risk of infection to encapsulated bacteria, such as *Streptococcus pneumoniae*, *Neisseria meningitidis*, and *Haemophilus influenzae* type b, Gram-negative pathogens, and intra-erythrocyte parasites [16].

Vaccination against *Streptococcus pneumoniae*, *Neisseria meningitidis*, and *Haemophilus influenzae* type b can help prevent overwhelming post-splenectomy infection by establishing immunological memory. Initial vaccinations should be administered 14 days before a planned splenectomy or 14 days after an urgent splenectomy to ensure an adequate response [16].

Splenectomy is not associated with an increased risk of viral reactivation; however, in this case, the presence of the invasive meningococcal disease may have triggered its reactivation. In fact, the neurotropism of HHV-6 is currently well known. HHV-6 can invade the brain during acute infection, when there is disruption of the blood-brain barrier, and can cause febrile seizures. Moreover, its genome has been shown to persist in the CNS, even in healthy adults without symptoms, as shown by PCR exams of tissues obtained at postmortem examination [4]. Therefore, in the face of a serious CNS infection, the reactivation of the virus will likely occur more easily. Furthermore, the presence of bacteremia and the prescription of intravenous corticosteroids may have contributed to a reduction in overall immunity.

Regarding the diagnosis, it was inferred from the results of PCR analysis of both serum and CSF. Similarly, in Schallhorn CS et al. diagnosis was also made based on PCR analysis of serum and CSF [15].

In our case, given the presence of active and concurrent CNS disease, the positive PCR analysis of both serum and CSF for HHV-6, and the retinal lesion characteristics suggestive of a viral etiology, we concluded that the ocular disease was most likely caused by HHV-6. *A posteriori*, the good response to treatment, was also a point in favor of this diagnosis. The absence of ocular hypertension, while not supporting a herpetic etiology, did not exclude it either.

*Streptococcus pneumoniae* should be considered as a cause of endophthalmitis, especially in patients with meningitis and bacteriemia associated with the microorganism. The authors did not consider this a pneumococcal endogenous endophthalmitis because the patient did not present with ocular pain, had only mild ciliary injection and mild vitritis, and had been receiving treatment with intravenous ceftriaxone since the first day of hospitalization.

Retinitis without significant vitreous infiltrate is more suggestive of a neurotropic infection such as those associated with the herpes family of viruses, rather than bacterial endophthalmitis. In the case of atypical or unfavorable progression, immediate collection of an aqueous or vitreous sample should have been performed. In our case, since no ocular sample was collected, the diagnosis remained presumptive.

The treatment of this type of infection is still a matter of debate. Similar to CMV, in vitro studies have shown that ganciclovir and foscarnet can effectively inhibit HHV-6 replication, while acyclovir has been found to be less effective. Insufficient response to acyclovir has been observed in clinical cases reported by Keorochana N. et al., Schallhorn SC. et al., and Maslin J. et al., where treatment was switched to ganciclovir/foscarnet, resulting in a successful outcome. However, there are also reports in the literature in which the infection was resolved with acyclovir/valacyclovir, as in Malamos P. et *al* [4, 6, 8, 10, 15].

The most frequent treatment scheme is intravenous ganciclovir 5 mg/kg/dose every 12 h for 2 weeks followed by oral valganciclovir 900 mg twice daily as a maintenance therapy. Several studies had variable maintenance periods, ranging from 4 weeks to 8 months [10, 11, 14, 15]. In our case, given the favorable clinical progression and the observed healing of the retinal lesion, we decided to discontinue oral therapy after 4 weeks.

In Maslin J. et al. the treatment was stopped on day 45 as PCR analysis for HHV-6 on a second CSF and aqueous humour samples were negative [8]. The decision to suspend treatment based on the PCR result must be cautious, as chromosomal integration may occur. Chromosomal integration signifies that the entire viral genome is incorporated into the host’s DNA. Typically, HHV-6 DNA is not present in the serum and/or plasma of healthy individuals, non-infected by HHV-6. Thus, when HHV-6 DNA is detected in plasma or serum, one might assume that there is active viral replication. However, in individuals with HHV-6 chromosomal integration, whose cells contain at least one copy of the HHV-6 genome, HHV-6 DNA loads of 10⁴ to 10⁵ copies/mL of plasma can be detected, as the simple act of drawing blood causes a certain amount of cellular lysis. If chromosomal integration happens, PCR becomes an unreliable method for monitoring treatment, as the number of DNA copies may not correlate with active viral replication. This event should be suspected if, given a good clinical evolution, the copy number remains high. This was a point that was raised in the clinical case of Bajric J. et al. [13, 18].

## Conclusion

In conclusion, we present a case of unilateral retinitis associated with HHV-6 in a splenectomised patient. The diagnosis was promptly presumed based on the detection of the virus in the serum and CSF, enabling the early initiation of appropriate treatment and contributing to the preservation of good visual acuity.

## Data Availability

No datasets were generated or analysed during the current study.

## Abbreviations

BCVA: Best corrected visual acuity
CMV: Cytomegalovirus
CNS: Central nervous system
CSF: Cerebrospinal fluid
EBV: Epstein-Barr virus
HIV: Human immunodeficiency virus
HHV-6: Human herpesvirus-6
PCR: Polymerase chain reaction
VSH1: Herpes simplex virus type 1
VSH2: Herpes simplex virus type 2
OD: Right eye
OS: Left eye
